# *In situ* compression of micropillars under coherent X-ray diffraction: a case study of experimental and data-analysis constraints

**DOI:** 10.1107/S1600576723000493

**Published:** 2023-02-28

**Authors:** Pierre Godard, Mariana Verezhak, Tarik Sadat, Florent Mignerot, Vincent L. R. Jacques, David Le Bolloc’h, Carsten Richter, Felisa Berenguer, Ana Diaz, Steven Van Petegem, Pierre-Olivier Renault, Ludovic Thilly

**Affiliations:** a Institut Pprime, CNRS – Université de Poitiers – ISAE ENSMA, 11 Boulevard Marie et Pierre Curie, Téléport 2, 86073 Poitiers Cedex 9, France; b Paul Scherrer Institut, Forschungsstrasse 111, 5232 Villigen, Switzerland; c Laboratoire de Physique des Solides, 1 rue Nicolas Appert, Bâtiment 510, 91405 Orsay Cedex, France; d European Synchrotron Radiation Facility, 71 Avenue des Martyrs, 38000 Grenoble, France; e Synchrotron SOLEIL, L’Orme des Merisiers, Saint-Aubin, BP 48, 91192 Gif-sur-Yvette, France; SLAC National Accelerator Laboratory, Menlo Park, USA

**Keywords:** coherent X-ray Bragg diffraction, micropillars, compression, *in situ*, InSb

## Abstract

Micropillar compression is a method of choice to understand mechanics at small scale. It is mainly studied with electron microscopy or white-beam micro-Laue X-ray diffraction. The aim of the present article is to show the possibilities of the use of diffraction with a coherent X-ray beam. InSb micropillars in epitaxy with their pedestals (*i.e.* their support) are studied *in situ* during compression. Firstly, an experiment using a collimated beam matching the pillar size allows determination of when the sample enters the plastic regime, independently of small defects induced by experimental artefacts. A second experiment deals with scanning X-ray diffraction maps with a nano-focused beam; despite the coherence of the beam, the contributions from the pedestal and from the micropillar in the diffraction patterns can be separated, making possible a spatially resolved study of the plastic strain fields. A quantitative measurement of the elastic strain field is nevertheless hampered by the fact that the pillar diffracts at the same angles as the pedestal. Finally, no image reconstructions were possible in these experiments, either *in situ* due to a blurring of the fringes during loading or post-mortem because the defect density after yielding was too high. However, it is shown how to determine the elastic bending of the pillar in the elastic regime. Bending angles of around 0.3° are found, and a method to estimate the sample’s radius of curvature is suggested.

## Abstract 2

The possibilities offered by three coherent X-ray diffraction experimental setups applied to micropillar compression are discussed here. The yield stress was precisely determined with a collimated beam. Methods to deconvolve the signal coming from the pillar from that coming from the pedestal and to estimate *in situ* the curvature radius of the micropillar are also proposed here.

## 1. Introduction

In 1924 it was observed that the tensile strength of wires increases when the diameter decreases in the micrometre and sub-micrometre range (Taylor, 1924 ▸). Since then, the yield stress and/or fracture stress of single crystals have been quantified as functions of the object size and crystal quality (Brenner, 1956 ▸; Uchic *et al.*, 2004 ▸; Gruber *et al.*, 2008 ▸; Richter *et al.*, 2009 ▸; Kiener *et al.*, 2011 ▸). However, several complementary methods are needed to fully understand the mechanical behaviours of micro-crystals. For example, *in situ* transmission electron microscopy offers insights on the plasticity mechanisms, like dislocation nucleation, escape at free surfaces or cross slip (Oh *et al.*, 2007 ▸, 2009 ▸); *in situ* scanning electron microscopy (SEM) allows determining the activated slip systems and their location (Kiener *et al.*, 2008 ▸; Thilly *et al.*, 2012 ▸), a set of information also provided by scanning Laue micro-diffraction; and the latter also gives clues on strain gradients and initial defect content (Maaß *et al.*, 2007 ▸; Kirchlechner *et al.*, 2012 ▸). The purpose of the present article is to illustrate how a relatively new technique, coherent X-ray diffraction (CXRD) (Vartanyants & Robinson, 2001 ▸), may be used to obtain insights on the deformation during the compression of a micropillar. The main difficulty lies in the fact that the micropillar and the pedestal diffract at the same angles; hence, three different experimental setups are presented and their complementarity is discussed.

In the Bragg geometry, CXRD is a nondestructive tool that is very sensitive to crystalline defects and strain gradients in samples from tens of nanometres to a few micrometres thick. When the acquisition allows retrieving the phase of the diffracted field, whether with a support constraint for a nano-particle [a technique called BCDI, for Bragg coherent diffraction imaging (Pfeifer *et al.*, 2006 ▸; Clark *et al.*, 2013 ▸; Labat *et al.*, 2015 ▸; Hofmann *et al.*, 2017 ▸)] or with scanning of a focused beam with a sufficient overlap between each adjacent step [*i.e.* Bragg ptychography (Godard *et al.*, 2011 ▸; Hruszkewycz *et al.*, 2012 ▸; Chamard *et al.*, 2015 ▸; Pateras *et al.*, 2015 ▸)], three-dimensional strain images with a resolution down to ∼10 nm may be obtained. Furthermore, due to its acquisition time being of the order of 10–30 min, BCDI allows *in situ* imaging of slow processes (Ulvestad *et al.*, 2015 ▸; Dupraz *et al.*, 2017 ▸). However, BCDI is limited to isolated objects, while Bragg ptychography requires long acquisition times that are problematic in terms of the setup stability, radiation damage and monitoring responses to external perturbations. A much more versatile method consists of directly comparing the diffraction patterns collected in CXRD with simulated data. Statistical information on the number of defects may be retrieved (Chamard *et al.*, 2008 ▸; Favre-Nicolin *et al.*, 2010 ▸; Jacques *et al.*, 2013 ▸), or, for a very small number of defects, their type may be determined (Jacques *et al.*, 2011 ▸; Dupraz *et al.*, 2015 ▸). Recently, it has also been used to investigate the elastic strain during tensile tests on nano-wires (Lazarev *et al.*, 2018 ▸; Shin *et al.*, 2018 ▸).

For demonstration purposes, we chose indium antimonide (InSb) single crystals, whose plasticity properties are known to greatly differ according to sample size (Kedjar *et al.*, 2010*a* ▸,*b* ▸). Like most III–V semiconductors, InSb is brittle at room temperature and atmospheric pressure in the bulk state, but exhibits ductile behaviour at the micro-scale (Thilly *et al.*, 2012 ▸). This is due to the plasticity mechanism: at room temperature InSb deforms via leading partial dislocations whose sources are activated only once. In the bulk state, the sources are not numerous enough to accommodate an applied strain larger than a few per cent, so macroscopic samples are brittle. As the size of the sample decreases, each dislocation nucleation event introduces a larger increment of plastic strain, and InSb pillars show a ductile behaviour when the diameter is smaller than ∼20 µm. In single-crystalline micropillars, it has been observed that the partial dislocations leave the sample, resulting in a band of isolated stacking faults that spread from top to bottom of the pillar (Thilly *et al.*, 2012 ▸). A consequence of the emission of a single dislocation per source is that there are no dislocation avalanches, and so no load drops or strain burst in the stress–strain curve. Compression tests on InSb micropillars are thus highly reproducible, and this material serves as a model system for the development of methodological tools for the study of plasticity at small scales (Wheeler *et al.*, 2016 ▸; Verezhak *et al.*, 2018 ▸).

**Figure 1:**
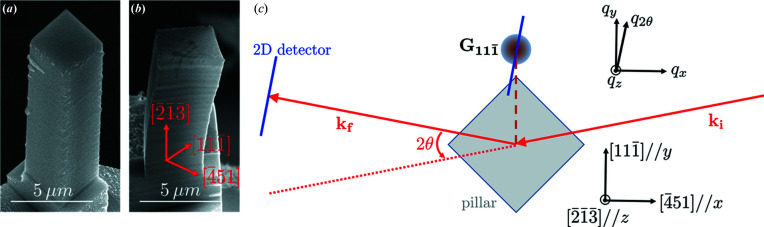
(*a*) Initial and (*b*) post-mortem SEM images of the 3.5 × 3.5 × 10.5 µm micropillar MP_3.5_ deformed at the Cristal beamline. The highest applied stress on the pillar was 570 MPa. (*c*) The scattering geometry with a scattering angle of 2θ = 22°, top view, where **k**_i_ and **k**_f_ are the incident and scattered wavevectors, respectively. The diffraction condition is 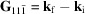 . The pixelated detector is positioned perpendicularly to **k**_f_.

The micropillars (MP) of the present study were prepared by Ga^+^ ion milling on single-crystalline InSb wedges. They have square cross sections and an aspect ratio of 3:1. Three samples were studied: MP_3.5_ for the experiment with the micro-beam, and MP′_2_ and MP′′_2_ for the experiments with the nano-focused beam. The subscript denotes the length (in micrometres) of the cross-section edge. SEM images of the virgin pillars are shown in Figs. 1 ▸(*a*) and 3(*a*). All the samples have the same crystallographic orientation, given in Fig. 1 ▸(*b*). This 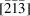 orientation induces slip in a single slip system.

The article is organized as follows. Section 2 shows results with a micro-beam. For reasons detailed below, no three-dimensional reciprocal-space maps could be analysed and we are reduced to studying sections of the Bragg peak. Then experiments with a nano-focused beam are reported: scanning X-ray diffraction microscopy (SXDM) in Section 3 and a method to quantify the elastic bending of the pillar under deformation in Section 4. Finally, we discuss the possibilities and the difficulties of *in situ* CXRD for micro- and nano-mechanics characterization. Additional information about sample preparation and mechanical tests is given in the supporting information.

## 2. Observation of very first defects with a micro-beam

The first experiment was performed at the Cristal beamline of the synchrotron radiation source SOLEIL, France. A monochromatic beam with a central energy *E* = 8.500 keV (corresponding wavelength λ = 1.459 Å and energy resolution Δ*E*/*E* = 1.4 × 10^−4^ leading to a longitudinal coherence length of about λ*E*/2Δ*E* ≃ 0.5 µm) was defined with a secondary source 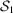 (200 × 100 µm large) (vertical times horizontal) at 13 m from the sample. Then slits 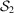 opened to 10 × 10 µm and positioned 0.2 m upstream of the sample location selected a portion of the beam approximately the size of the micropillar. To evaluate the range of incident angle characterizing the X-ray beam, we give the Fresnel numbers *N*_F_ = *a* ^2^/4λ*r* associated with the slits, where *a* is the slit aperture and *r* is the propagation distance; it is generally admitted that Fraunhofer diffraction (or the far-field regime) occurs when 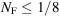 (Goodman, 2004 ▸). For the slits 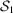 , we have *N*_F_ ≃ 5.3 in the vertical direction and *N*_F_ ≃ 1.3 in the horizontal direction, so we can suppose that the field incoming on the slits 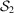 is well collimated. For these slits, we have *N*_F_ ≃ 0.9 at the sample position. Hence, despite the presence of Fresnel fringes, the incident beam may still be considered as being parallel.

Our micro-compression device imposed that the micropillar was placed vertically. The first studied sample was MP_3.5_ with *L* × *W* × *H* = 3.5 × 3.5 × 10.5 µm and the crystalline orientation given in Fig. 1 ▸(*c*).

The 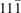 Bragg peak was followed *in situ* during the compression tests. The diffracting planes were vertical, which allows an easy interpretation of the directions of the peak shifts (see below). The incident beam was at θ_B_ = 11.23° from the 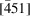 direction; the small value of θ_B_ limited the attenuation due to polarization. A Maxipix detector was placed 2.15 m after the sample. It contains 516 × 516 square pixels of 55 µm size. The Fresnel number associated with the propagation from the sample to the detector is *N*_F_ ≃ 0.02 [with *a* = 3.5(2)^1/2^ µm], which is well into the far-field regime.

The pillar and the pedestal, being milled from the same single-crystalline wedge, both diffract at the same incident and diffraction angles. Furthermore, due to the size of the micropillar and the micro-beam, the latter was simultaneously diffracted by the pillar and by its pedestal. However, when loading, one knows that the pillar deforms significantly more than the pedestal because of the cross-section difference. The Poisson effect induces an increase of the probed lattice parameter 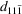 and the diffracting planes may rotate. As shown in Fig. 1 ▸(*c*), the horizontal direction *q*_2θ_ on the detector is close to the 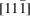 direction and thus approximately probes the lattice parameter evolution. The vertical direction is parallel to 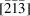 ; a shift of the peak along that direction corresponds to a bending of the pillar around the axis 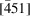 . The Bragg peak 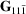 can also move along *q*_*x*_, associated with a twist (or torsion) around the vertical direction 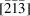 .

Performing traditional rocking curves with a scan of the incident angle ω was impossible under load: any rotation (like any translation) would induce large vibrations on the sample that would immediately lead to the sample failure. Hence, the twist was monitored with energy scans, but with a limited range. Finally, the 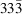 reflection caused by a harmonic of the monochromator adds another peak overlapping the peak of interest. We are thus limited here to a qualitative description of the micropillar behaviour during the compression test.

**Figure 2:**
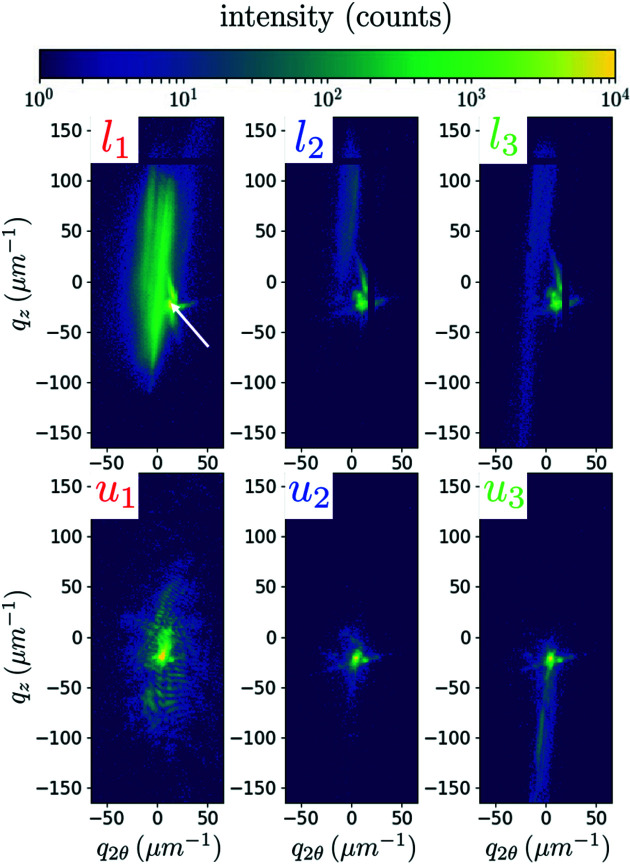
Collected diffraction patterns with a micro-beam at 8.500 keV on MP_3.5_, under load (*l*_*i*_) and after the subsequent retraction (*u*_*i*_). Three loads are shown: *l*_1_ = 330 MPa, *l*_2_ = 530 MPa and *l*_3_ = 570 MPa. The white arrow on *l*_1_ points to the peak of the pedestal. The coordinates *q*_2θ_ and *q*_*z*_ correspond to the horizontal and vertical directions on the detector, respectively. The intensity scale is limited to 10 000 photons to reveal the diffuse intensity, but the maximum intensity was actually ∼53 000 photons. The acquisition time was 0.5 s.

Fig. 2 ▸ shows diffraction patterns at some points of a load–unload series, at a fixed energy of 8.500 keV and with an acquisition time of 0.5 s. The three loading states of *l*_1_, *l*_2_ and *l*_3_ were observed for applied forces of 4000, 6500 and 7000 µN, corresponding to stress values of 330, 530 and 570 MPa, respectively. As we will see, these applied forces cover the full regime, from the elastic regime, to the first signs of plasticity (most probably at the top of the pillar), and finally to the plastic regime. The applied force of 7000 µN was the highest load applied to this pillar. For each load *l*_*i*_, we also show the state *u*_*i*_ that is the unloaded state just after *l*_*i*_.

The unloaded state *u*_1_ displays a typical coherent diffraction pattern with well defined fringes. They are separated by an average value of 5.5 µm^−1^, which corresponds to 1.15 µm in the sample space. We assume that this distance is due to the Fresnel fringes in the incident beam. However, it is striking how the speckles are blurred under load (compare *l*_1_ with *u*_1_ in Fig. 2 ▸). This shows how the coherence of the recorded diffraction signal is degraded while compressing the sample, even in the elastic regime. This complete blurring of the fringes appears as soon as a few tens of micronewtons are applied to the micropillar. We attribute this to tiny vibrations induced by the compression tip or to small defects that move during the loading; if these defects move sufficiently fast, the measured diffraction pattern is an incoherent sum of the different configurations that occur during the integration time. Hence, with this setup, CXRD does not seem appropriate for studying a continuous loading, and the load–unload series is necessary.

Another observation comes from the peak splitting under load, seen in *l*_1_, *l*_2_ and *l*_3_. As explained above, this splitting along *q*_2θ_ is induced by the change of lattice parameter in the 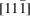 direction. The elongated peak on the left-hand side of the patterns corresponds to the compressed micropillar. The pillar peak is at lower 2θ values, which corresponds to larger 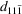 values. The more localized peak is the pedestal contribution, whose very large section almost prevents any deformation at this force value. As a consequence, the pedestal peak does not significantly change for the different loads (from *l*_1_ to *l*_3_), remaining very compact and displaying the characteristic diffraction pattern of a large and perfect crystal illuminated by an almost square-shaped X-ray beam. As we might expect, the peak associated with the pillar changes under these small loads: it broadens in the vertical direction, evidencing a bending. This is clearly reversible, as monitored in *u*_1_ and *u*_2_. Finally, the pillar peak appears much fainter in *l*_2_ and *l*_3_ than in *l*_1_. This is due to a slight sample misorientation (torsion) from the perfect diffraction condition, a particularly stringent condition when the incident beam is parallel.

When unloading to the *u*_1_ or *u*_2_ states, the pillar and pedestal peaks overlap again, showing that, overall, the pillar returns to its initial crystallographic orientation. Up to 530 MPa (state *u*_2_), the patterns do not change drastically in the unloaded state. However, the intensity decreases and the fringes are lost, which is attributed to the appearance of small crystalline defects or to the slow drift of the setup. Conversely, a clear streak along *q*_*z*_ occurs after the loading at 570 MPa (state *u*_3_) due to coordinated plastic defects stored in the pillar. The post-deformation SEM image shown in Fig. 1 ▸(*b*) confirms that *u*_3_ corresponds to an unloaded state after an excursion into the plastic regime: while the virgin pillar has straight faces, it clearly presents irreversible deformation in the post-mortem image. The SEM observation angle allows one to detect an obvious bending, but the twist is visible as well.

The main advantages of this setup are (*a*) that illuminating the whole pillar at once allows for a quick characterization of the behaviour of the sample, which can be useful in experiments where the initial response of the sample to an external perturbation must be determined; (*b*) that the entire compressed crystal and the reference crystal (the pedestal) are simultaneously diffracting on the detector, the pedestal being a good reference to track the displacements of the diffraction peak from the pillar; and (*c*) that a highly collimated beam is very sensitive to lattice strain. The corresponding disadvantages are twofold. First, looking at the whole pillar may lead to difficulties in interpreting the diffracted signal, *e.g.* the defects appearing at the top of the pillar due to a slight misalignment between the sample and the compression tip may hide the volume defects that the test aims at uncovering. Second, the detector arm was mechanically connected to the sample stage, hence prohibiting detector movement under load, since the resulting vibrations would have been fatal to the pillar. Hence, it is not only classical rocking curves that are impossible but also energy scans with sufficient ranges: when the energy changes, the diffraction angle shifts and so does the pattern on the detector, allowing only a very small region in reciprocal space to be probed. For these reasons, we complemented this experiment with another *in situ* CXRD beam time, this time with a nano-focused beam, and with a decoupled detector arm, so that its position could be adjusted at each step of a scan in beam energy. The results are presented in the next two sections.

## 3. Separation of pedestal and pillar peaks

**Figure 3:**
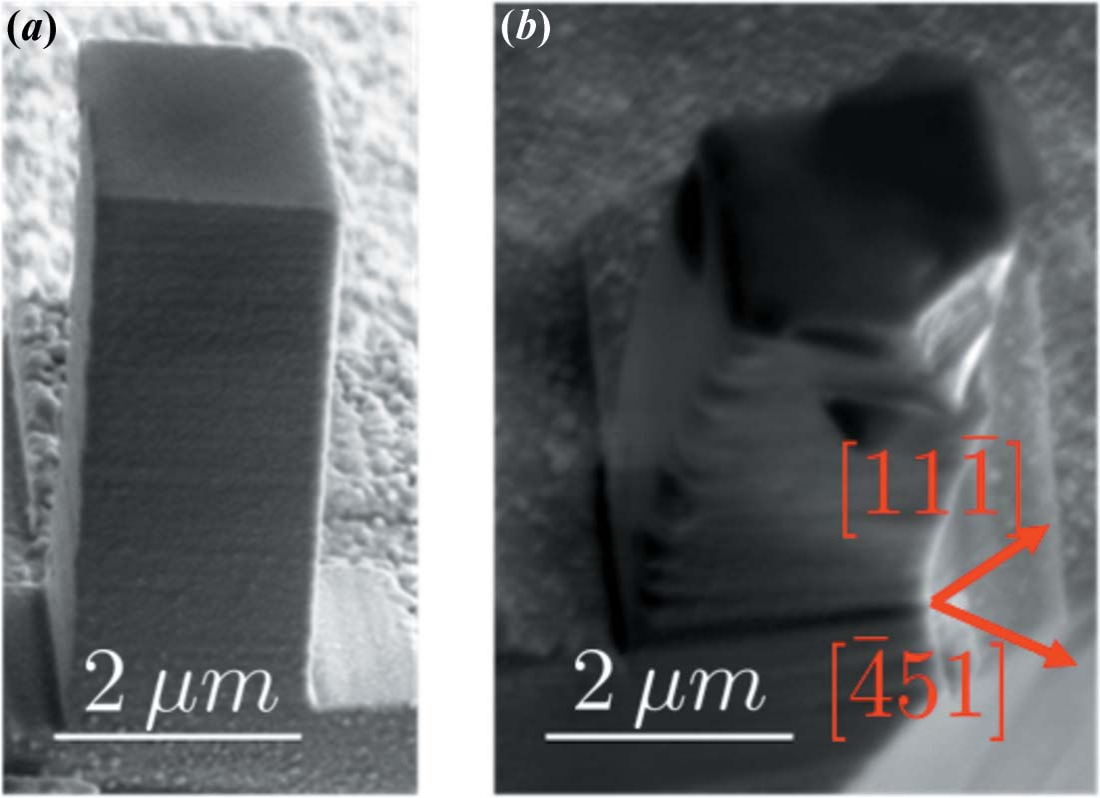
SEM images of the micropillar MP′_2_, 2 × 2 × 6 µm, studied with nano-diffraction at ID01, (*a*) before and (*b*) after deformation. The highest applied stress on the pillar was 610 MPa. The pillar axis is 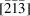 .

With the pedestal being tens of micrometres thick, its volume is so large compared with the micropillar that its diffraction signal is often overwhelming even if it is illuminated only with the tail of a nano-focused beam centred on the pillar. To allow a separation of the pillar and the pedestal contributions, we performed nano-diffraction on a highly deformed pillar (*in situ* and post-mortem energy scans and post-mortem SXDM). This pillar, MP′_2_, *L* × *W* × *H* = 2 × 2 × 6 µm, was loaded up to 2440 µN, and the force–displacement curve showed that yielding occurred at ∼2000 µN, corresponding to 500 MPa, confirming the value obtained for the pillar studied in the previous section. As a check, we can clearly see that the pillar is deformed from top to bottom in the post-mortem image of Fig. 3 ▸. In this image, we also observe that cracks have appeared at the pillar’s top.

Nano-beam X-ray diffraction measurements were carried out at beamline ID01 of the European Synchrotron Radiation Facility (ESRF), France. The beam energy of 8.000 keV was defined by an Si(111) double-crystal monochromator with a resolution of Δ*E*/*E* = 1.4 × 10^−4^. For beam focusing, a pair of bendable Kirkpatrick–Baez mirrors were used, which are achromatic optics and therefore allow energy scanning without changing the optics. The optics aperture was reduced to 200 and 60 µm in the vertical and horizontal directions, respectively, to match the coherence lengths of the beam. Two-dimensional forward ptychography on a well characterized test object (a Siemens star) reconstructed the X-ray probe profile and determined a beam size of ∼235 × 385 nm [vertical times horizontal, full width at half-maximum (FWHM) of the intensity] at the focal position (see Fig. S.M.1 of the supporting information). The setup for the sample holder and micro-compression machine was similar to the one used at the Cristal beamline. The Maxipix detector was mounted 1.41 m downstream of the sample, on a detector arm that is independent of the sample rotational and translational stage. As for the sample MP_3.5_, the 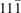 diffraction peak was probed.

**Figure 4:**
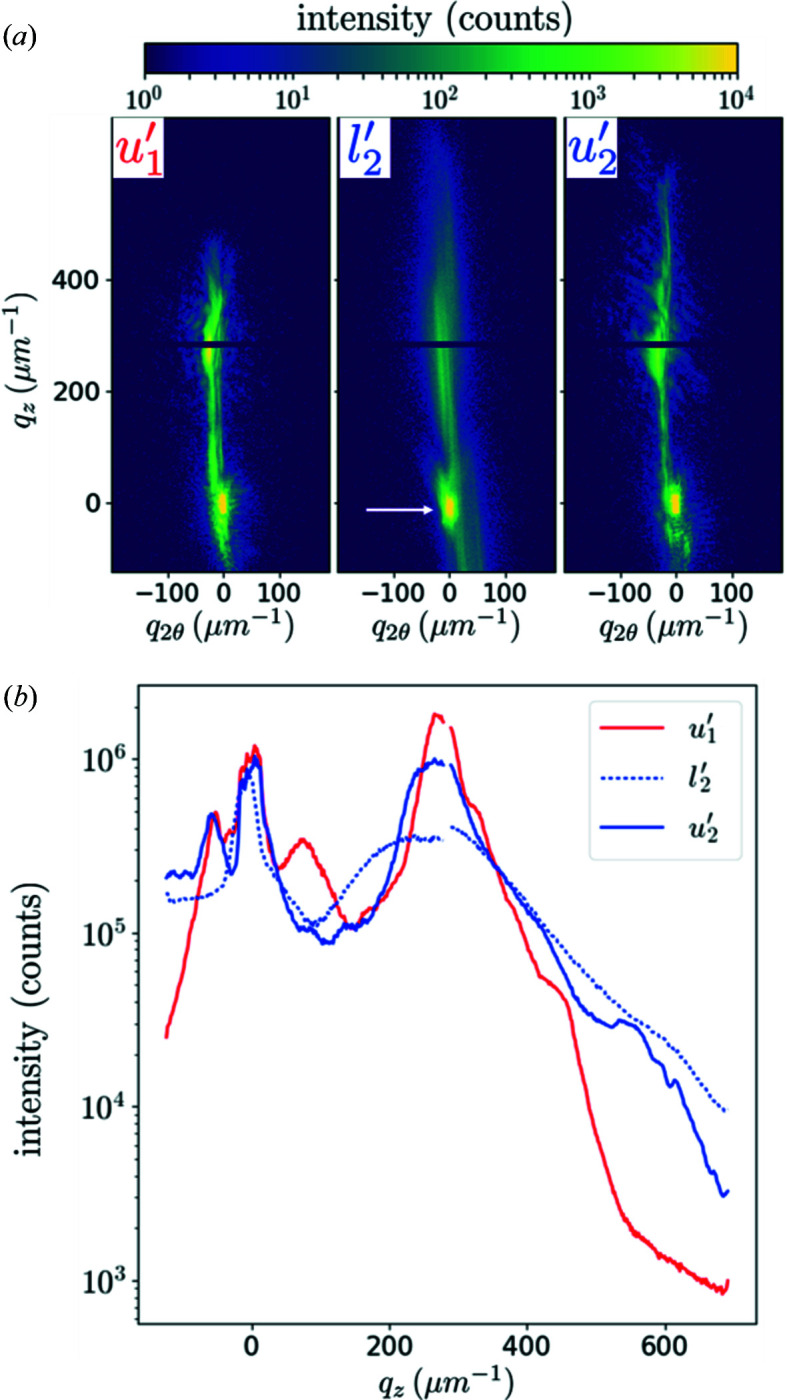
(*a*) Diffraction patterns of MP′_2_ at the centre of the rocking curves after 550 MPa (*u*′_1_), under 610 MPa (*l*′_2_) and after 610 MPa (*u*′_2_). The logarithmic scale is from 1 to 10 000 but the pedestal peak (white arrow) hits more than 60 000 counts. The acquisition time was 1 s. (*b*) Corresponding intensities integrated along *q*_*x*_ (probed with energy scans) and *q*_2θ_.

Fig. 4 ▸(*a*) shows a diffraction pattern at an unloaded state *u*′_1_, which occurs after a loading to 2200 µN (550 MPa). The beam was positioned at the centre of the pillar. We see a small spot towards the bottom of the detector and an intense tail. This tail shows at least one secondary peak; since this peak is observed after retraction of the compression tip, it corresponds to an irreversible bending of the pillar, that is to a reorientation induced by plasticity.

**Figure 5:**
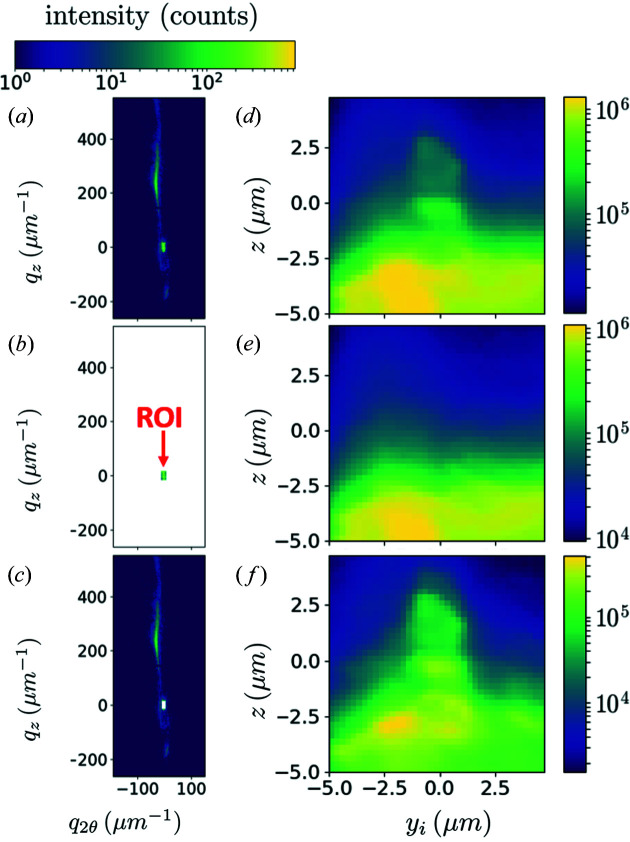
Post-mortem diffraction patterns of MP′_2_ and nano-diffraction maps (a pixel on the map is the intensity integrated on a selected region of the detector), after 610 MPa (state *u*′_2_). (*a*)–(*c*) A diffraction pattern collected at the centre of the pillar with an acquisition time of 0.02 s. (*a*) On this pattern we select areas on the detector: (*b*) an ROI and (*c*) the complementary part. (*d*)–(*f*) Corresponding integrated intensity maps, showing that the ROI is associated with the pedestal only. The coordinate *y*_*i*_ describes the horizontal direction perpendicular to the beam propagation direction. The colorbar above (*a*) applies to (*a*), (*b*) and (*c*), with white meaning masked area.

If the load increases to 2440 µN (state *l*′_2_, 610 MPa), we recover what was observed in Fig. 2 ▸: the fringes are completely blurred under load. Other streaks appear post-mortem (state *u*′_2_), evidencing a multiplication of defects. The force of 2440 µN is the highest load applied on MP′_2_, so the SEM image in Fig. 3 ▸(*b*), as well as the SXDM map in Fig. 5 ▸, corresponds to the *u*′_2_ state.

Three-dimensional reciprocal-space maps were obtained by performing energy scans from 7.800 to 8.600 keV, with a step of 5 eV and a per-step acquisition time of 1 s. During the energy scan, the detector was moved in order to keep the main peak on the same pixel, so that the resulting set of images is similar to a rocking scan (Lauraux *et al.*, 2020 ▸). The integration along *q*_*x*_ and *q*_2θ_ is shown in Fig. 4 ▸(*b*). The peak at the bottom of the detector (taken as the reference *q*_2θ_ = *q*_*z*_ = 0) does not move, whether under load or after retraction (states *u*′_1_, *l*′_2_ and *u*′_2_), a first indication that it corresponds to the pedestal. On the other hand, the secondary peak close to *q*_*z*_ = 275 µm^−1^ is vastly broadened under load (curve *l*′_2_), suggesting that it corresponds to the pillar. Furthermore, the fact that this peak is weak on the detector (state *u*′_2_) but intense after integration shows that it is broad along *q*_*x*_.

To check this hypothesis, we performed post-mortem nano-diffraction on this sample, in a mesh of 41 × 41 points with a 250 nm step and an acquisition time of 0.02 s. The SXDM procedure developed at ID01 was used (Chahine *et al.*, 2014 ▸). We could not perform SXDM under load because moving the compression device while scanning would have broken the sample. A typical diffraction pattern is shown in Fig. 5 ▸(*a*), once again corresponding to the micropillar centre. On this pattern, we selected a region of interest, hereafter called ROI, of 20 × 10 pixels, shown in Fig. 5 ▸(*b*). This ROI has an area of 32 × 16 µm^−1^, associated in the sample space with ∼200 × 400 nm, which is the extension of the illumination on the sample. The integrated intensity maps (at the centre of the rocking curves), shown in Fig. 5 ▸(*d*) for the whole detector and in Fig. 5 ▸(*e*) for this ROI, confirm that the ROI corresponds to the pedestal: excluding the ROI in the analysis of the integrated intensities increases the contrast of the pillar [Figs. 5 ▸(*c*) and 5 ▸(*f*)]. Hence, though intensities do not simply add in CXRD, a separation of the two scattering intensities is possible because the signal from the (very large and unstrained) pedestal has a very small extension in reciprocal space.

The patterns shown in Figs. 4 ▸ and 5 ▸, whether under load or after retraction, are too distorted for a description of the strain field in the sample to be possible with CXRD. Hence, in the next section, we concentrate on small applied forces, where strain gradients are much smoother.

## 4. *In situ* elastic deformation observations and simulations with a nano-focused beam

**Figure 6:**
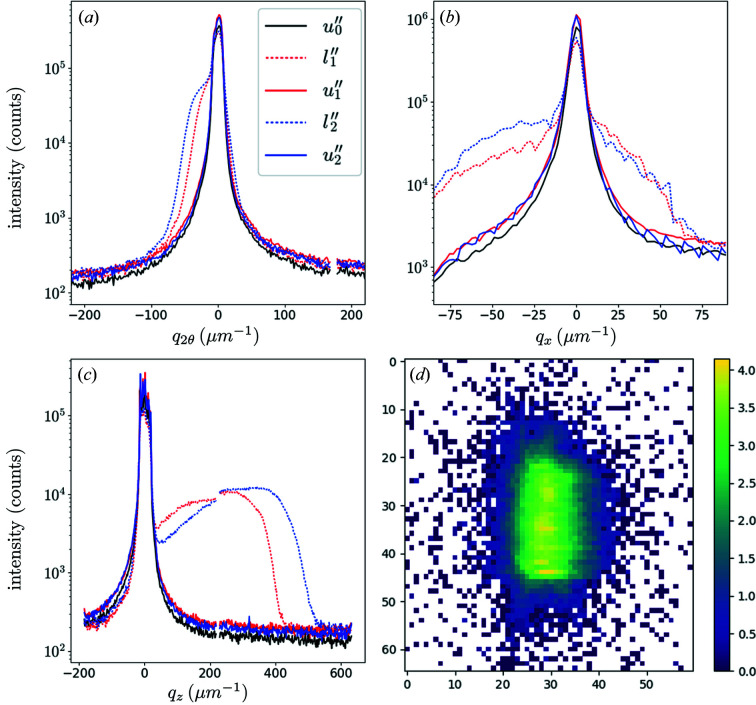
Integrated diffraction intensities of the micropillar MP′′_2_, characterizing (*a*) the elastic strain, (*b*) the twist and (*c*) the bending around 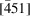 . The direction *q*_*x*_, parallel to 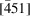 , is probed with energy scans, while *q*_2θ_ and *q*_*z*_ are the horizontal and vertical directions on the detector, respectively. Furthermore, *u*′′_0_ is before any loading, while *u*′′_1_ and *u*′′_2_ are after applied stresses of 130 and 190 MPa, respectively. The horizontal and vertical scales are adapted for each part. (*d*) The diffraction pattern associated with *u*′′_0_.

For the micropillar MP′′_2_ (same size as MP′_2_), our aim was to remain in the elastic regime. More precisely, we first applied a load up to 750 µN, then one load to 500 µN, denoted by *l*′′_1_, and a second to 750 µN, *l*′′_2_. Once again, *u*′′_*i*_ is the unloaded state of the pillar just after the loaded state *l*′′_*i*_. The reference state was obtained before any loading and is denoted *u*′′_0_ [Fig. 6 ▸(*d*)]. A two-dimensional scan showed the pillar homogeneity at this stage. The beam-to-sample position was then fixed and five energy scans from 7.800 to 8.200 keV with 5 eV steps were performed corresponding to the states *u*′′_0_, *l*′′_1_, *u*′′_1_, *l*′′_2_ and *u* *′′*_2_. The loads 500 and 750 µN correspond to 130 and 190 MPa, respectively, *i.e.* stress values much lower than the elastic limit.

Reciprocal-space maps were recorded with an acquisition time of 1 s per diffraction pattern. The beam was centred on the pillar. Fig. 6 ▸ shows the intensity integrated along different directions: in (*a*) we integrated the measured three-dimensional reciprocal-space maps along *q*_*x*_ and *q*_*z*_, in (*b*) the integration is along *q*_2θ_ and *q*_*z*_ (equivalent to rocking curves), and in (*c*) the integration is along *q*_*x*_ and *q*_2θ_. The superposition of the curves corresponding to the unloaded states of *u*′′_0_, *u*′′_1_ and *u*′′_2_ confirms that the whole test remained in the elastic regime. In these states, the widths of the peaks are in qualitative agreement with the size of the scattering volume; this volume is defined by the beam size in the horizontal direction (∼400 nm), the pillar thickness along the beam path [∼2(2)^1/2^ µm] and the beam size in the vertical direction (∼200 nm), respectively. In the loaded states, the signal coming from the micropillar is characterized by a broadening of the curves; it is associated with a shoulder showing a larger lattice spacing along 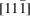 (Poisson effect) [Fig. 6 ▸(*a*)] and a range of orientations evidencing a twist around 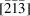 [Fig. 6 ▸(*b*)] and a bending around 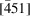 [Fig. 6 ▸(*c*)]. In the following, we present a method to quantify this bending.

As we probe the Bragg peak 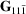 , we are only sensitive to the projection of the displacement field along this direction, *i.e.* to *u*_*y*_ (see Fig. 1 ▸). We will denote by *u*_*y*_(*x*), *u*_*y*_(*y*) and *u*_*y*_(*z*) the functions that correspond, in the absence of shear, to the twist, the strain and the bending, respectively. The bending component shown in Fig. 6 ▸(*c*) is thus described by *u*_*y*_(*z*), and we approximate it by a low-order polynomial, *u*_*y*_(*z*) = β_1_ *z* + β_2_ *z* ^2^. There is no constant term in this expression as constant displacements do not produce any change on the diffraction patterns. It is thus set to 0 for convenience. The linear term is measured directly as the shift 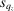 of the pillar peak’s centre of mass. From Fig. 6 ▸(*c*), we estimate 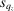 to be ∼200 µm^−1^ for *l*′′_1_ and 250 µm^−1^ for *l*′′_2_. This corresponds to mean bending angles of 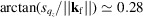 and 0.35°, respectively (where **k**_f_ is the scattered wavevector), in agreement with values obtained for similar InSb micropillars compressed *in situ* during micro-Laue experiments (article in preparation) and other compression experiments of germanium micropillars.

The bending heterogeneities will now be characterized with the second-order term in *u*_*y*_(*z*). For this, we use a simple model relating the width of the peak to the radius of curvature of the pillar under load. First, as the attenuation length in InSb at this energy is 7.26 µm^−1^, the absorption of the beam in the pillar is neglected. Then, we approximate the incident field with a (phase free) Gaussian beam, 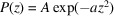 ; Fig. S.M.2 shows that this is a reasonable approximation with *a* such that the FWHM of the beam amplitude is 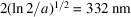 . Given this beam extension, the pillar is supposed to be infinite along the vertical direction. In this case, analytical results are possible for the Fourier transform.

Explicitly, at the exact diffraction condition, the phase of the effective electron density is 

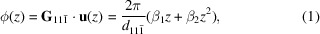

but the first-order term only shifts the diffraction pattern (determined previously) and has a negligible impact on the radius of curvature. Hence, we write ϕ(*z*) = *bz* ^2^, where *b* = 2πβ_2_/*d*_111_, so that the scattered far-field is 

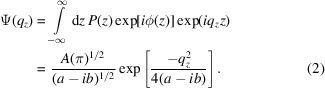

With *b* as the only free parameter, we fitted the diffraction patterns so that the scattered intensity *I*(*q*_*z*_) = |Ψ(*q*_*z*_)|^2^ matches the measured intensity.

**Figure 7:**
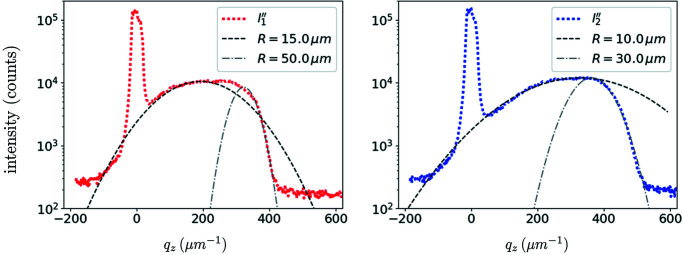
A fit of the bending of micropillar MP′′_2_ under compression, with applied stresses of 130 MPa (left) and 190 MPa (right).

The radius of curvature of the function 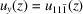 is, by definition, 

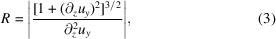

so that *R* ≃ 1/2β_2_. The results are plotted in Fig. 7 ▸. The first comment is that only orders of magnitudes may be obtained: indeed, this simple model cannot catch the asymmetry of the peak. In other words, even at these small loads, higher-order terms in the Taylor development of the strain field are needed to correctly describe the observations; we tried third- and fourth-order polynomials but did not find good matches for the whole curves. A possible explanation is that the bending is heterogeneous over the beam size. The bending radius for the *l*′′_1_ state lies between 15 and 50 µm. Of course, the more loaded state is associated with smaller radii, between 10 and 30 µm for the *l*′′_2_ state.

Two effects may explain these surprisingly low values. First, the divergence of the beam has been neglected. Though the different modes show relatively flat fronts in the principal lobe, a slightly divergent beam would spread the intensity in reciprocal space, and would be interpreted here as small radii of curvature. The second effect that may explain the small values of *R* is that only a small region of the 6 µm tall pillar was explored. This region is approximately given by the beam FWHM (235 nm). Hence, the radii estimated here may not be representative of the bending of the whole pillar. A next step would be to scan the pillar along its axis, taking advantage of the fact that loading in the elastic regime is a reversible process.

## 5. Discussion and conclusions

Firstly, despite the misalignments between the flat punch and the micropillar, the compression tests are reproducible in the sense that the yield stress of the pillar deformed *in situ* at Cristal was between 530 and 570 MPa, while it was ∼500 MPa for the pillar deformed at ID01. Moreover, the samples considered in this study belong to a small range of diameters (2 to 3.5 µm edge length) and all have the same aspect ratio (3:1), so that no size effects are expected. Hence, we can suppose that we are studying several states of the same test.

Some difficulties usually encountered in compression of micropillars were limited in this study because of the small applied strains; these include sink-in of the pillar into the pedestal, friction at the interface between the flat punch and the pillar, and stress heterogeneities at the pillar’s edges. However, the load–unload series may have impacted the dislocation nucleation and escape, with the vanishing applied stress regularly imposed on the pillar; this applied stress may even get into slight tension during retraction due to an adhesive layer on the top of pillar caused by the preparation process. The main issue is probably the misalignment between the sample and the flat punch. This probably led to the pillar MP′_2_ being damaged, as seen in Fig. 3 ▸. Moreover, Maaß *et al.* (2009 ▸) showed with micro-Laue diffraction and with crystal-plasticity finite element simulations (FEMs) that misalignments imply that the first activated slip system may not be the geometrically predicted one.

CXRD on micropillars obtained by focused-ion-beam milling of a bulk sample has several complications: (i) for *in situ* studies, it is impossible to scan or to tilt the sample under load; and (ii) the pedestal, which needs to be as large as possible to avoid deformation, diffracts at the same angles as the micropillar. Item (i) has been partially solved with energy scans rather than rocking curves, as already performed by Cornelius *et al.* (2011 ▸, 2012 ▸), Cha *et al.* (2016 ▸) and Richard *et al.* (2020 ▸); however, like in any scanning diffraction method (Kirchlechner *et al.*, 2012 ▸; Shin *et al.*, 2018 ▸), it is impossible to move the sample with respect to the beam under load so that only one beam-to-sample position can be probed for *in situ* studies (this does not prevent, of course, shifting the optics whenever possible). In this respect, the use of coherent beams should partially circumvent this problem, since the long-range position of the scatterers is encoded in the diffracted field.

The fact that the pedestal diffracts at the same angle as the object of interest [item (ii)] may be viewed as a difficulty or as an advantage. On the one hand, its signature on the diffraction patterns overwhelms the micropillar contribution, especially after small loads like *u*_1_ in Fig. 2 ▸ (where the whole pillar was illuminated) or *u*′′_1_ in Fig. 6 ▸ (where the nano-beam was ∼3 µm above the pedestal); this will obviously lead to difficulties if imaging is intended, particularly prior to any deformation. On the other hand, we have shown that it is limited to a small area on the detector, and it can be used as a reference, like a reference powder in classical X-ray diffraction. We refer the reader to Diaz *et al.* (2010 ▸) for a detailed discussion on a related example.

More generally, most characterization methods have to deal with a trade-off between strain sensitivity (*i.e.* resolution in reciprocal space) and resolution in sample space, as reviewed and quantified by Schülli & Leake (2018 ▸). This is often associated with the beam collimation or smallness trade-off. With a micro-beam defined by slits close to the sample (Section 2) or a nano-focused beam (Sections 3 and 4), we explored very different possibilities. In the first case (Cristal setup), we showed that we can distinguish isolated defects from coordinated defects representative of the tests. In the second case (ID01 setup), we showed that quantitative nano-diffraction imaging with SXDM maps is not easy due to the overwhelming presence of the pedestal peak, even when the beam is in the top part of the 6 µm tall micropillar. However, this was only at the centre of the rocking curve; the contribution of the pedestal will rapidly decay out of the exact Bragg position. Furthermore, we showed how CXRD may be used to estimate the radius of curvature of the micropillar under compression. The next step should be to interpret the peak asymmetries. Concerning the resolution–sensitivity trade-off, we refer the reader to the article by Verezhak *et al.* (2021 ▸) for a new characterization method that aims at winning on both sides: a plane wave illuminates the micropillar but a pinhole defines the exit-field extension, allowing for ptychographic reconstructions at tens of nanometres resolution with a high strain sensitivity.

In this article, we have considered a purely quadratic phase to reproduce the curvature of the pillar, allowing one to obtain a quantitative bending value. More advanced information on the bending or twisting of the sample may be obtained using an analytic solution of elasticity theory (like the buckling column formula in our case). For example, Lazarev *et al* (2018 ▸) succeeded in imaging the native state in a ∼1.5 µm long area of a GaN nano-wire, and then used *in situ* CXRD to characterize the strain field of the device under operation. Because the mere fact of depositing metallic contacts induced too much strain for successful reconstructions, they acquired three-dimensional CXRD reciprocal-space maps that give clues on the strain level in the nano-wire, after contact deposition and while applying voltage bias. FEMs allowed them to estimate the ultimate tensile strength and one piezoelectric coefficient of the nano-wire. Post-mortem FEMs also appear, among others, in the work of Cornelius *et al.* (2012 ▸) to check the shape of the reciprocal-space maps, in the work of Hruszkewycz *et al.* (2012 ▸) to separate the lattice strain observed by Bragg projection ptychography in an SiGe-on-SOI (silicon-on-insulator) structure into its different origins, in the work of Dupraz *et al.* (2017 ▸) to determine the three-dimensional displacement field from one of its components imaged with BCDI and boundary conditions, and in the work of Dzhigaev *et al.* (2017 ▸) to evaluate a plastic relaxation parameter in an InGaN/GaN core–shell nano-wire.

However, all these simulations can be quite lengthy; it would be particularly time consuming in our case, since the misalignments are unknown and would have to be guessed by trial and error. Finally, the bending-angle estimation presented here could be obtained on-line on a synchrotron radiation source.

The examples considered in this article show that CXRD allows an approximate estimation of the elastic strain field in a micro-object under compression thanks to a comparison with simulations. The type of defects could probably be identified with reciprocal-space maps on slightly deformed pillars. Moreover, CXRD may allow a phasing of the diffraction patterns to obtain an image of the strain field. In our case, we acquired three-dimensional Bragg ptychography data sets but we did not succeed in obtaining any reliable reconstruction. Three reasons can explain the inefficiency of the reconstruction: the contribution of the pedestal mentioned above, the thickness of the pillar, which is large with respect to the longitudinal coherence length, and the initial crystalline quality. Examples with a few dislocations have already been presented, but it is generally accepted that CXRD will apply only for low defect density. We think that it will be difficult to obtain information on individual defects with CXRD on these samples: on virgin crystals, the scattered intensities from the micropillar and from the pedestal overlap; shortly after the yield point, there are too many defects to be interpreted with CXRD. The more interesting route we envision is to gather statistical information, probing reflections sensitive to the stacking faults and reflections that are not, as previously done by Chamard *et al.* (2008 ▸), Favre-Nicolin *et al.* (2010 ▸) and Hill *et al.* (2018 ▸).

## 6. Related literature

The following additional references are cited in the supporting information for this article: Swygenhoven & Petegem (2010) ▸. 

## Supplementary Material

Supporting information. DOI: 10.1107/S1600576723000493/te5107sup1.pdf
