# Supplementary material for: In situ compression of micropillars under coherent X-ray diffraction: a case study of experimental and data-analysis constraints
Source: J Appl Crystallogr. 2023 Feb 28;56(Pt 2):381–90. doi: 10.1107/S1600576723000493 (PMC10077849; doi:10.1107/S1600576723000493)
Supplement: Supplementary file 1 [file j-56-00381-sup1.pdf]

JOURNAL OF
APPLIED
CRYSTALLOGRAPHY

Volume 56 (2023)

Supporting information for article:

In situ compression of micropillars under coherent X-ray diffraction: a case study of experimental and data-analysis constraints

Pierre Godard, Mariana Verezhak, Tarik Sadat, Florent Mignerot, Vincent L.R. Jacques, David Le Bolloc'h, Carsten Richter, Felisa Berenguer, Ana Diaz, Steven Van Petegem, Pierre-Olivier Renault and Ludovic Thilly

In situ Compression of Micropillars under Coherent X-Ray Diffraction : a Case Study of Experimental and Data Analysis Constraints

PIERRE GODARD,* MARIANA VEREZHAK, TARIK SADAT, FLORENT MIGNEROT, VINCENT L.R. JACQUES, DAVID LE BOLLOC'H, CARSTEN RICHTER, FELISA BERENGUER, ANA DIAZ, STEVEN VAN PETEGEM, PIERRE-OLIVIER RENAULT AND LUDOVIC THILLY

. *E-mail: pierre.godard@univ-poitiers.fr*

1. Sample preparation

InSb single crystal was selected for the study (S-doped with $n = 4.88\,10^{16}$ cm^{-3}). A wedge with $< 123 >$ orientation was cut to allow the fabrication of a row of isolated micropillars with $< 123 >$ orientation to promote the activation of only one dislocation slip system ($< 111 >$ primary slip plane with a Schmid factor $m = 0,47$). Micropillars were milled at the top of the wedge to provide access to individual synchrotron X-ray diffraction analysis; this geometry imposes to fabricate micropillars with a square section by milling perpendicularly to their axis with an angle of $\pm 45°$. Micropillars were designed to have an aspect ratio (height to lateral dimension) of 3 and were milled on top of a square pedestal. InSb micropillars were fabricated using a Focused Ion Beam (FIB) Helios NanoLab G3 CX from ThermoFischer Scientific equipped with a gallium source. A high current of 2.5 nA was used to obtain a rough cut of the individual pedestals and additional

steps were used to mill the micropillars on their pedestals. The current was gradually reduced until the final polishing step at 40 pA (the voltage was kept constant at 30 kV throughout the process). A height adjustment was performed on each micropillar in order to comply with the above-mentioned aspect ratio.

2. Mechanical testing

The samples were compressed uniaxially with the micro-compression device described in (Maaß *et al.*, 2007) or (Swygenhoven & Petegem, 2010) designed in-house at Paul Scherrer Institute. The compression head is a truncated conical diamond indentation tip with an end tip radius of 11 μm. The device was initially calibrated based on "in-air" compression. The compression axis was chosen to be along $[\bar{2}\bar{1}\bar{3}]$ crystallographic direction, which corresponds to the pillars vertical axis. The loading and unloading rates were 6.7 μN/s.

3. Supplementary Figures

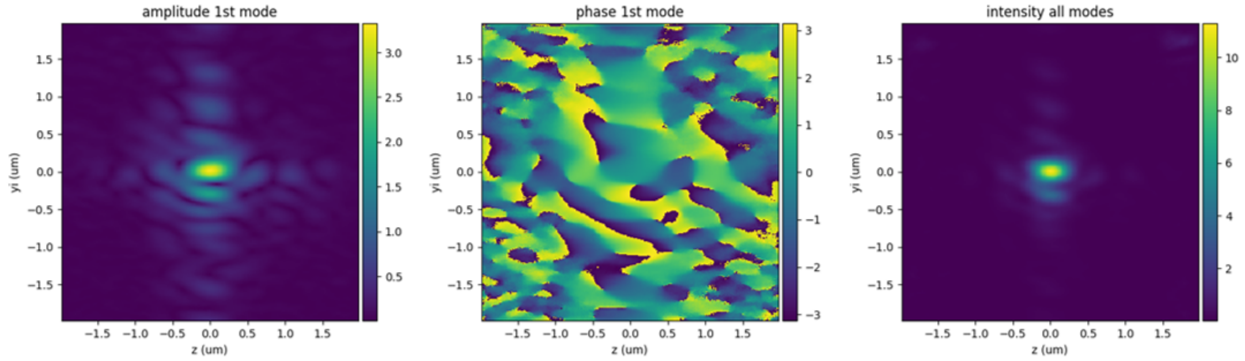

Fig. S.M.1. Reconstructed probe at ID01 beamline on a test object (Siemens star), with forward two-dimensional ptychography. Left and middle : the principal mode is shown, it had a contribution of 66%. The phase is flat in the first lobe. Right : intensity when all modes are taken into account. z is the vertical coordinate.

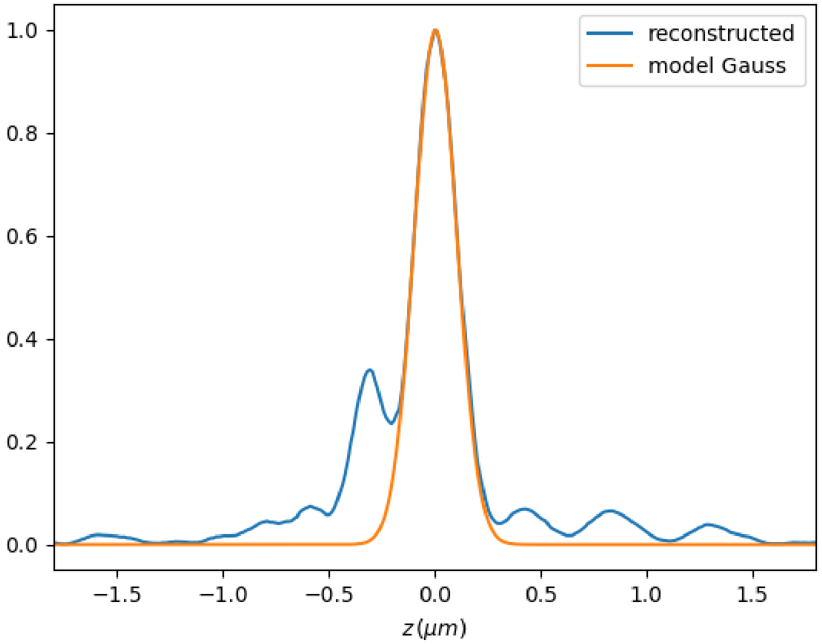

Fig. S.M.2. Vertical section of the incident beam intensity at ID01 and fit with a Gaussian beam.

References

Maaß, R., Petegem, S. V., Swygenhoven, H. V., Derlet, P. M., Volkert, C. A. & Grolimund, D. (2007). *Phys. Rev. Lett.* **99**, 145505.

Swygenhoven, H. V. & Petegem, S. V. (2010). *JOM*, **62**, 36–43.
